# Development of a Self-Help Web-Based Intervention Targeting Young Cancer Patients With Sexual Problems and Fertility Distress in Collaboration With Patient Research Partners

**DOI:** 10.2196/resprot.5499

**Published:** 2016-04-12

**Authors:** Jeanette Winterling, Maria Wiklander, Claire Micaux Obol, Claudia Lampic, Lars E Eriksson, Britta Pelters, Lena Wettergren

**Affiliations:** ^1^Division of NursingDepartment of Neurobiology, Care Sciences and SocietyKarolinska InstitutetHuddingeSweden; ^2^Center of HaematologyKarolinska University HospitalStockholmSweden; ^3^Stress Rehabilitation ResearchDepartment of Clinical Sciences Danderyd HospitalKarolinska InstitutetStockholmSweden; ^4^Medical Management CenterDepartment of Learning, Informatics, Management and EthicsKarolinska InstitutetStockholmSweden; ^5^Department of Infectious DiseasesKarolinska University HospitalStockholmSweden; ^6^School of Health SciencesCity University LondonLondonUnited Kingdom; ^7^School of Health and WelfareHalmstad UniversityHalmstadSweden

**Keywords:** adolescent, clinical trial, Internet, neoplasms, online systems, patient participation, technology, telemedicine, young adult

## Abstract

**Background:**

The Internet should be suitable for delivery of interventions targeting young cancer patients. Young people are familiar with the technologies, and this patient group is small and geographically dispersed. Still, only few psycho-educational Web-based interventions are designed for this group. Young cancer patients consider reproductive health, including sexuality, an area of great importance and approximately 50% report sexual problems and fertility-related concerns following cancer treatment. Therefore, we set out to develop a self-help Web-based intervention, Fex-Can, to alleviate such problems. To improve its quality, we decided to involve patients and significant others as research partners. The first 18 months of our collaboration are described in this paper. The intervention will subsequently be tested in a feasibility study followed by a randomized controlled trial.

**Objective:**

The study aims to describe the development of a Web-based intervention in long-term collaboration with patient research partners (PRPs).

**Methods:**

Ten former cancer patients and two significant others participated in building the Web-based intervention, using a participatory design. The development process is described according to the design step in the holistic framework presented by van Gemert-Pijnen et al and evaluates the PRPs’ impact on the content, system, and service quality of the planned intervention.

**Results:**

The collaboration between the research group and the PRPs mainly took place in the form of 1-day meetings to develop the key components of the intervention: educational and behavior change content, multimedia (pictures, video vignettes, and audios), interactive online activities (eg, self-monitoring), and partial feedback support (discussion forum, tailored feedback from experts). The PRPs influenced the intervention’s content quality in several ways. By repeated feedback on prototypes, the information became more comprehensive, relevant, and understandable. The PRPs gave suggestions concerning the number of exercises and pointed out texts and pictures needing revision (eg, experienced as normative or stereotypical) to increase the persuasiveness of the program. The system quality was improved by PRPs’ feedback on design, technical malfunctions, and navigation on the website. Based on feedback about availability of professional support (technical problems and program content), the organization for support was clarified, which increased service quality. The PRPs also influenced the research project on an overall level by suggesting modifications of inclusion criteria for the RCT and by questioning the implementation plan.

**Conclusions:**

With suggestions and continuous feedback from PRPs, it was possible to develop a Web-based intervention with persuasive design, believed to be relevant and attractive for young persons with cancer who have sexual problems or fertility distress. In the next step, the intervention will be tested in a feasibility study, followed by an RCT to test the intervention’s effectiveness in reducing sexual problems and fertility distress.

**Trial Registration:**

International Standard Randomized Controlled Trial Number (ISRCTN): 36621459; http://www.isrctn.com/ISRCTN36621459 (Archived by WebCite at http://www.webcitation.org/6gFX40F6T)

## Introduction

There is a need for psycho-educational interventions adapted for adolescents and young adults with cancer [1,2]. Web-based interventions are presumed to be a useful mode to deliver such interventions as young people are well accustomed to the technologies [1] and the patient group is relatively small and geographically dispersed. The Internet has proven to be effective for both delivery of information [3,4], support [5,6] and psychological treatment [7] for a wide range of health problems; such delivery is known as eHealth [8]. However, Internet interventions also face problems such as high dropout rates and non-usage during the test phase as well as after implementation [9,10]. Collaboration with patients in the development of Web-based interventions has been suggested to make the technology more attractive and user friendly, thereby improving uptake and impact of the intervention [10].

### Patient and Public Involvement

Patient and public involvement in research [10] is regarded as an integral part of good scientific practice [11] and is increasingly requested from research funders. A recent systematic review showed that patient and public involvement has beneficial effects on all stages of the research process [12]. Different approaches to involvement exist. Consultation is when end users are asked for their views and these views are used to inform decision making. Collaboration involves active, ongoing partnership with end users where both collaborating parties share decisions about the research. In user-controlled research, the end users rather than the professionals have the power and the initiative to carry out the research. Long-term collaboration with end users has been proposed to increase the relevance, quality, and validity of eHealth interventions [13,14]. Still, patient and public involvement has, with few exceptions [9], been limited to the consultation level and involved end users only on single occasions [15-17]. Patient and public involvement has seldom been applied in the development of Internet interventions to be tested in randomized controlled trials (RCTs) [12]. In this study, a collaboration level of patient and public involvement was used, in preparation for a subsequent RCT.

### The Fertility and Sexuality Following Cancer (Fex-Can) Project

Previous studies from our research group [18-20] and others [21] have shown that adolescents and young adults diagnosed with and treated for cancer have concerns about fertility and sexuality. It is also well known that sexual problems and reproductive issues often are neglected in clinical care [22,23] and that care providers lack training for such discussions [22,24]. Meta-analyses have found Web-based interventions to be effective in the areas of sexual [25] and reproductive health [26]. Based on the above findings, we set out to develop a self-help Web-based intervention, Fex-Can, to alleviate sexual problems and fertility-related distress in young people treated for cancer.

The effectiveness of the intervention will be evaluated in an RCT embedded in a nationwide cohort study directed towards individuals aged 16-40 in Sweden with selected cancer types, 1 year post-diagnosis. During 1 year, potential participants will be identified through national cancer registers and invited to participate in the cohort study. After consenting, participants will complete standardized questionnaires measuring sexual function and fertility distress (online or paper version). Those rating high levels of sexual dysfunction and/or fertility distress at baseline will be invited to participate in the RCT (closed user group trial) with two arms, testing the Fex-Can intervention versus control group. The Regional Ethical Review Board in Stockholm has approved the study. In Sweden, health care is mainly tax-funded and all Swedish citizens receive health care at limited costs [27]. The project, including development of the intervention followed by a feasibility study and an RCT, is financed by research grants. If the intervention is shown to be effective, it is planned to be implemented in regular care.

### The Holistic Framework for Development of eHealth Technologies

This study is based on the holistic framework for developing eHealth technologies by van Gemert-Pijnen et al [10] and focuses primarily on the design step of the model, that is, the co-creative participatory process of building the Web-based intervention. An essential principle in this framework is end users’ involvement throughout the development process. End users in this study were represented by former cancer patients and significant others, hereafter referred to as patient research partners (PRPs), who participated in repeated evaluation cycles of the eHealth technology. According to theory, the quality of an eHealth intervention can be evaluated on three different levels: content, system, and service quality [10,28]. Content quality includes creating information that is understandable, meaningful, and persuasive. System quality means that the technology is safe, user-friendly, and easy to manage. Service quality entails providing an e-service that is adequate and reliable, that is, providing prompt and empathetic support to participants regarding technical and general issues.

The study aims to describe the development of a Web-based intervention in long-term collaboration with PRPs.

## Methods

### Recruitment of Patient Research Partners

The PRPs were recruited from a previous study investigating sexuality and fertility among childhood cancer survivors [18], and through cancer nurse navigators at a university hospital. The research group set out to recruit women and men, aged 16-40, who had undergone cancer treatment for any of the cancer types selected for the planned RCT. In addition, we wanted to recruit a few significant others (partners or parents of young patients). In total, 13 PRPs were recruited—11 individuals previously diagnosed with cancer (2-9 years earlier) and 2 mothers of teenagers who had undergone cancer treatment agreed to participate in a 5-year long collaboration. The PRPs were all born in Sweden but lived in different parts of the country. All were fulltime working or studying with a majority having a university degree. The former patients were 7 women and 4 men, aged 20-41, and included singles as well as partnered individuals of whom 2 had children and 1 became a parent during our collaboration. The following cancer diagnoses were represented: Hodgkin lymphoma (n=5), tumors of the central nervous system (n=2), breast cancer (n=2), testicular cancer (n=2), cervical cancer (n=1), and Ewing sarcoma (n=1). One PRP (former patient) decided to leave the collaboration after attending one meeting, while the other 12 PRPs remained in the group.

The PRPs had a status of research partners rather than research participants and were paid for their participation in project meetings and time working with assignments. Additionally, PRPs were reimbursed for travel expenses and if needed, accommodation, as some traveled to meetings from distant places in Sweden.

### Project Management

The research group included researchers, health care providers, and a project coordinator with academic backgrounds in medicine, psychology, psychiatry, sociology, nursing, and arts. Their clinical backgrounds included primary care, psychotherapy, and counseling in cancer care and sexually transmissible infections, respectively. The composition of the research group altered during the 18-month period described, with an average of 6-7 researchers present at each meeting with the PRPs. The researchers had weekly project management meetings and regular contact with a network of professional collaborators including physicians and nurse practitioners in cancer and reproductive care, and sexual therapists. The research group was responsible for managing the collaboration with the PRPs, including strategic and logistic planning of meetings with PRPs. The researchers were also responsible for documenting and implementing PRPs’ ideas and for decisions regarding the scientific process.

A software company was contracted to build the Fex-Can Internet portal. Additionally, we collaborated with a Web designer, an illustrator, and a photographer. One of the research group members was responsible for contacts with the software company, and another team member acted as main contact person for the PRPs throughout the process.

### Forms for Collaboration

The collaboration mainly took place in the form of 1-day meetings. Efforts were made to establish a trustful collaboration between the researchers and the PRPs. All meetings included a joint lunch for all involved PRPs and researchers at the expense of the project.

**Figure 1:**
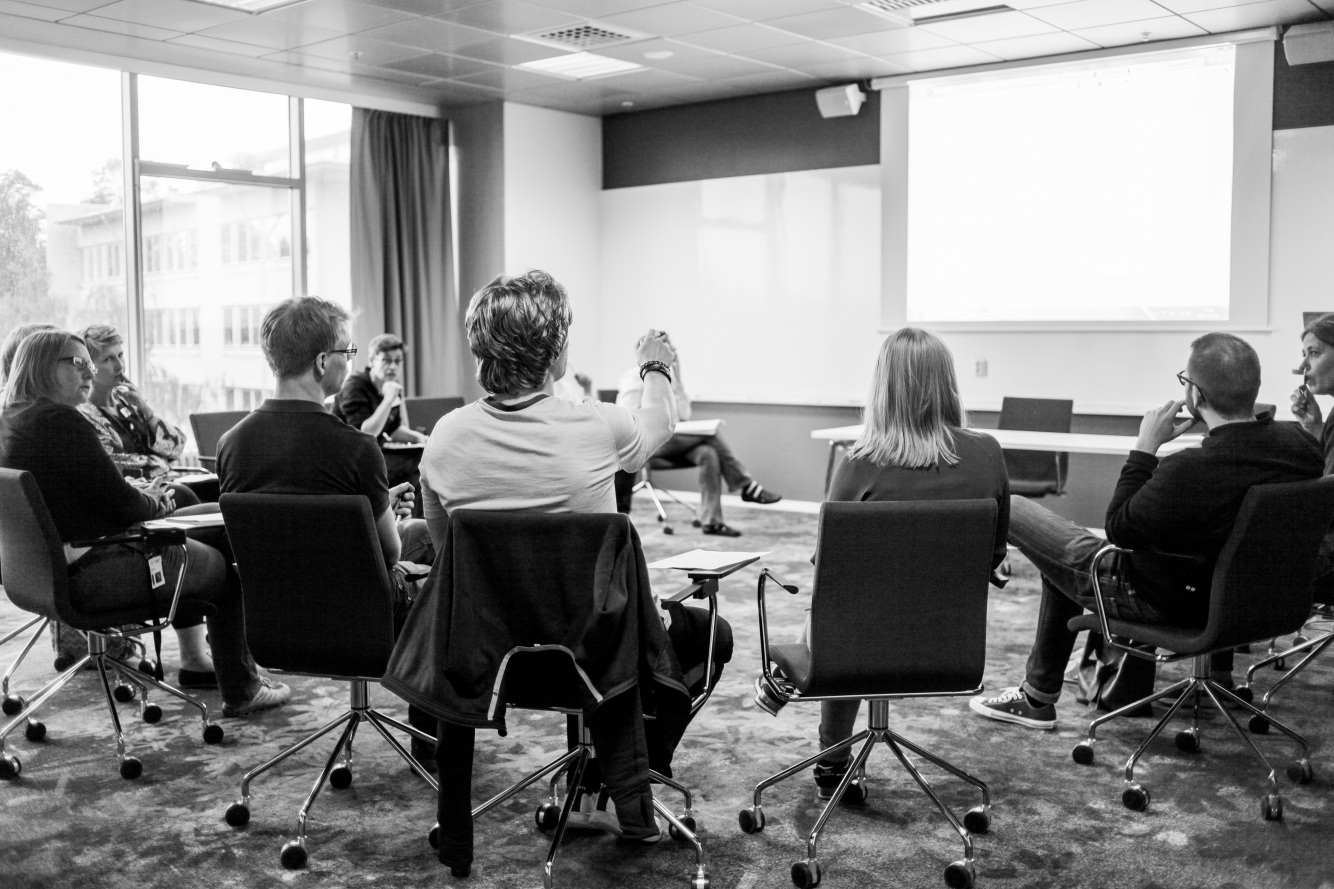
Plenary discussion at PRP meeting.

During the first meeting, the forms for collaboration between researchers and PRPs were agreed upon. Different forms of collaboration were discussed, for example, Web-based discussion forums for different age groups, video conferences, or physical meetings. The PRPs preferred to have meetings in person on a regular basis as 1-day get-togethers during weekends (PRP meetings), while communication between these meetings was to be carried out by email. The 1-day meetings included plenary (see Figure 1) and small group discussions as well as individual assignments. At subsequent meetings, the forms for collaboration were revisited and PRPs were asked if they wanted to continue to work in the same way. This procedure generated four additional PRP meetings within the design step; PRPs attended a median of four of the total five PRP meetings.

### Documentation

The results in this article are based on several sources of data. First, notes were taken by 2 members of the research team during each 1-day meeting with the PRPs. These notes covered all topics discussed and all opinions raised by the PRPs. The notes were compiled and presented at research group meetings when the notes were adjusted according to the impressions of all research group members. Minutes from all meetings, together with notes from contacts with PRPs between meetings were continuously compiled into a log book. Second, all researchers met directly after each 1-day meeting with the PRPs and reflected upon their impressions throughout the day. These reflections were added to the log of the collaboration with the PRPs. Third, the notes from previous meetings were also discussed with the PRPs to check that their opinions had been correctly understood. Fourth, the PRPs gave confidential feedback on content, layout, and functionality on some of the modules of the intervention directly into the Fex-Can portal.

The results in this article are based on this documentation and will be presented according to the quality criteria suggested by the holistic framework by Gemert-Pijnen et al [10]: content, system, and service quality. Furthermore, the process of building the intervention will be described, as well as the PRPs’ impact on the overall research project.

### Predefined Components of the Web-Based Intervention

Some features were planned to be included in the intervention, prior to the recruitment of patient research partners. According to key components for Internet interventions defined by Barak [29], these features were educational and behavior change content, multimedia (eg, pictures, video vignettes, and audios), interactive online activities (eg, self-monitoring), and partial feedback support (eg, discussion forum, tailored feedback from experts). The behavior change content was intended to convey a balance between problem solving (change) and acceptance strategies, including mindfulness. An aim was to affect participants’ autonomy (sense of control over one’s life), competence (perceived efficacy), and relatedness (“I am not alone with these problems”) [30]. However, details of the intervention were not planned and an objective for the collaboration was to let the PRPs have an impact on the composition of the content and structure of the intervention, to make it more relevant and attractive for users.

## Results

### Building the Intervention

**Figure 2:**
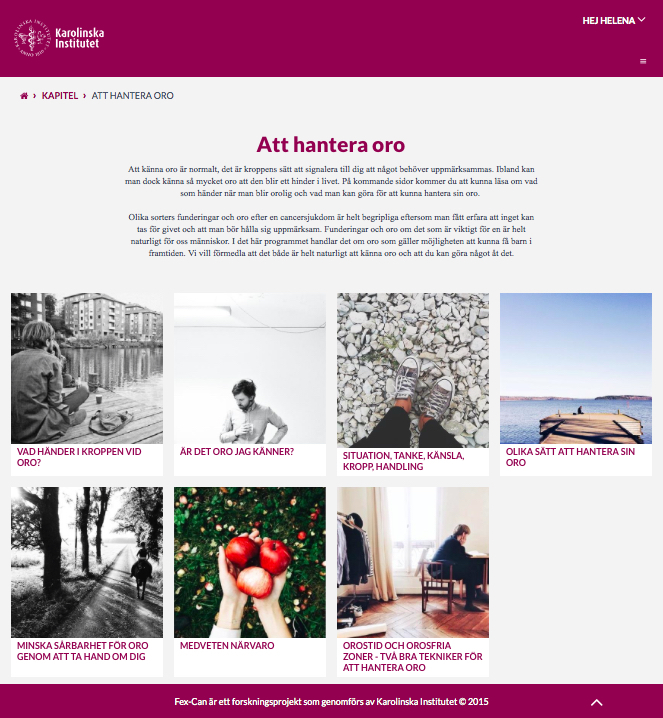
Example of module in the fertility stream of the intervention.

**Figure 3:**
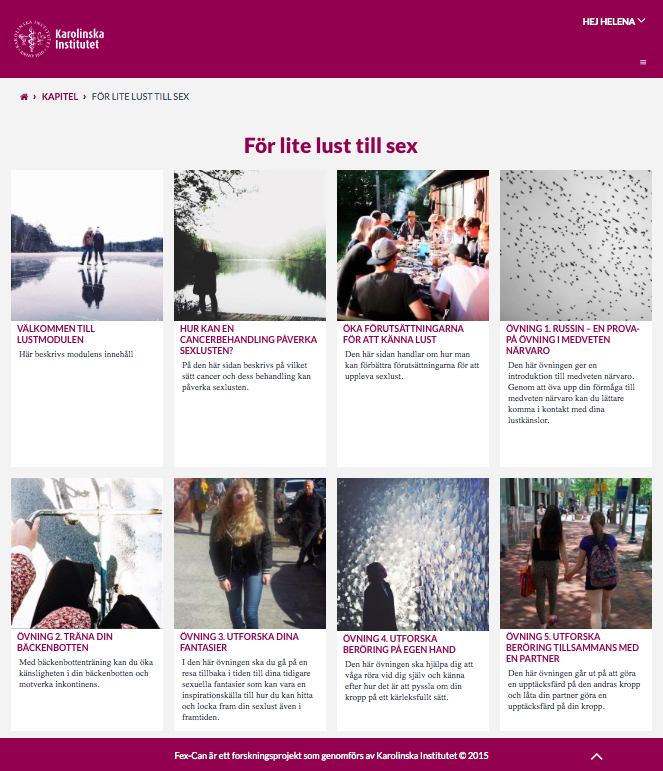
Example of module in the sexuality stream of the intervention.

In the first meeting, PRPs received information about the aims and framework of the planned study, and basic ethical principles in research. We also spent time on getting to know each other; the PRPs and the researchers introduced themselves and the PRPs shared their “cancer story.” For the second meeting, a mock-up of the Fex-Can Internet portal was created, based on existing knowledge regarding Web-based interventions, and presented to the PRPs. The mock-up suggested content divided into different modules and other functions such as expert and discussion forums. After discussing the planned set-up of the intervention, a prototype was produced for the following meeting. The researchers refined the Fex-Can intervention several times and the revised versions were discussed at meetings so that the PRPs were able to contribute to the process. Working materials, such as topic-relevant websites, suggested contents of specific modules, and access to different preliminary versions of a module, were mailed to the PRPs to be read before scheduled meetings. The Fex-Can intervention came to include several features organized in subsequent modules and divided in two streams: fertility and sexuality. Examples of two module overview webpages are presented in Figures 2 and 3. The following paragraphs describe how the PRPs had an impact on the intervention’s content quality, system quality, service quality, and overall project.

### Patient Research Partners’ Impact on Content Quality

During the first meetings, the PRPs expressed an overall concern that information on the website could cause emotional distress, especially in relation to information on risks of infertility and relapse of disease. At the same time, the importance of offering accurate and evidence-based detailed information was underscored. The views of what kind of information might be perceived as distressing differed within the group of PRPs. Following further discussion, we agreed that the Fex-Can intervention would convey a hopeful and encouraging attitude and present examples and strategies for dealing with problems, that its, it would be empowering. The PRPs wanted information on the website to be tailored to meet participants’ diverse needs regarding the amount of information, which made us organize the information on several levels, where participants have the option to read extended text.

The PRPs wished for the intervention to include more facts on sexuality and fertility related to side effects of specific cancer treatments as well as information about what side effects or symptoms were to be expected. All of this was added. Furthermore, the PRPs stressed the importance of including content related to bodily changes or body image in the sexual stream of the Fex-Can, which resulted in the addition of a separate module focusing on this aspect.

The PRPs shared their opinions about what types of exercises they thought should be included and how much time participants would be willing to spend on an exercise. They thought there were too many exercises and recommended us to carefully select a reduced number to be included and present all of them as optional. In particular, the included mindfulness exercises were debated since some PRPs were skeptical and questioned if the technique was evidence-based. After being presented with facts about the effects of such exercises followed by thorough discussions, the PRPs were supportive of including mindfulness in the Fex-Can intervention. Furthermore, PRPs appreciated that some misconceptions about mindfulness were disentangled on the website. In later versions of the intervention, the PRPs expressed that the included exercises appeared useful and reliable.

Another issue stressed by the PRPs was the importance of using an inclusive, easily comprehensible language matching a broad group of end users, including individuals with cognitive difficulties (common when diagnosed with brain tumors). A challenge was to use language without jargon, neither too colloquial, nor too formal. The PRPs stressed this necessity during earlier stages of working with the texts and expressed their satisfaction with the comprehensibility of the texts in the later versions of the website, indicating that a well-balanced language level had been established.

The PRPs emphasized that the content of the program should communicate an awareness of participants’ cancer experience. One part of this was the PRPs’ wish that the program should include other cancer patients’ stories to increase relatedness to others, for example, “I’m not the only one having these problems.” The researchers asked if the PRPs were willing to share their own stories (in text or as videos), which 5 agreed to do. The importance of choosing appropriate photos for the website was also emphasized by the PRPs. The photos should be representative also for participants who were still under treatment and troubled by side effects, for example, include persons with visible signs of cancer treatment such as hair loss, overweight/underweight, and scars.

In order to persuade participants in the intervention to stay in the program, the researchers suggested that a new module would be introduced every 2 weeks for participants in the intervention. This was supported by the PRPs who thought participants would be curious to see the next module. The PRPs also supported the idea that participants would receive feedback on what they had done so far in the program. Based on this, a timeline was included on the opening page of the intervention to visualize the progression of time during the 12-week program. Following suggestions made by PRPs to increase active participation in the intervention, weekly email reminders were also added as a feature in the program. Another topic discussed was the possibility of including quizzes in the program since they are interactive and have the potential to increase a sense of progressing. However, since some of the PRPs perceived such quizzes as stressfully competitive, quizzes were not included in the program.

A way to increase social dynamics in the program was to incorporate a discussion forum in the intervention as well as a counseling feature in the sexuality stream of the intervention, which also was supported by the PRPs. The feasibility of having a discussion forum with participants in such a wide age span as 16-40 years was thoroughly discussed, since persons of different ages might think differently about fertility and sexuality. After reflecting on advantages and disadvantages of dividing the forum into age groups, it was decided to keep one discussion forum but to create discussion threads for different age groups.

The PRPs emphasized that text and pictures included in Fex-Can should not be overly normative when relating to sexual problems and fertility distress. This includes addressing a diversity of sexualities, ethnicities, relationships, and ways of building a family instead of exclusively presenting white heterosexuals in monogamous relationships having biological children of their own. Initially, PRPs expressed that the intervention focused too much on couples and did not give enough attention to the potential problem of finding a partner. Therefore, the intervention was developed to better reflect the situation of singles, and a module on “How to meet a partner” was included in the sexual stream of the intervention.

### Patient Research Partners’ Impact on System Quality

The layout of the website was frequently discussed with the PRPs. They stressed that young people have high expectations on a website and that a design that is perceived as unprofessional would risk increasing participant dropout of the RCT. Researchers and PRPs agreed on the idea that the website should have a responsive design, that is, it should adapt its layout to various devices such as computers, smartphones, or tablets. PRPs also considered the Web address/domain to be an important factor that should communicate a rigorous and valid source, preferably university-based. The PRPs recommended using a more professional design than the first mock-up and suggested using a light background color, black text with classic font, and header in Karolinska Institutet’s promotional color. Furthermore, they wanted pictures to be included only if they had a function related to the text and not merely for esthetic purposes. A Web designer was consulted to make a neater layout with a uniform style and a better structure throughout the intervention, since the PRPs repeatedly stressed that the website was difficult to navigate. During the entire development process, the website had several technical malfunctions, which were pointed out by the PRPs. These problems were continuously adjusted in collaboration between the project coordinator and the software developer.

### Patient Research Partners’ Impact on Service Quality

The PRPs repeatedly stressed that technical malfunctions on the website were very frustrating. This increased the researchers’ awareness of the importance of almost instant support when intervention participants experienced technical problems. A plan for implementation of technical support within the intervention was added.

PRPs had objections to the suggested rules for the planned discussion forum. The PRPs expressed concerns regarding our plan to check all postings before they were published to prevent inappropriate texts and topics. This was believed to negatively affect the communication in the discussion forum because participants would lose interest if postings were delayed. Therefore, we decided that postings would be instantly visible in the forum but the researchers would be responsible for continuously checking for (and deleting) potentially inappropriate postings (or comments) within every 24 hours. Further, the PRPs stressed that replies to postings in the feature “ask an expert” should not take too long. Therefore, it was decided that the researchers would be responsible for the contact with external experts to make sure postings were replied to within 3 workdays. Likewise, it would be problematic if other website functions would not work properly. A Web-based support (e-service) was added within the program, where participants could ask researchers any type of questions (technical, content, and/or how to use the partial feedback support) via mail and get a fast reply. This e-service was planned to be given by someone from the research group who would understand and know all parts of the intervention and would be able to give this e-service in an emphatic way. Furthermore, a telephone support run by research group members was planned to be available for the website.

### Patient Research Partners’ Overall Impact on the Research Project

#### Naming the Project

After thoroughly discussing the purpose of the intervention, most of the PRPs did not appreciate the initial name of the project (“Life Interrupted”). They thought that the program should focus on moving forward in life rather than on the interruption in life that a cancer diagnosis might constitute. Several suggestions were discussed between the researchers and the PRPs, resulting in the more neutral name “Fex-Can, Fertility and sexuality following cancer”.

#### Plan for Evaluation and Implementation

In the beginning of the collaboration, the PRPs emphasized the need for the program to improve the care of cancer patients. They expressed that they would have appreciated access to such a program during and after their own cancer treatment, whether they had problems or not. Therefore, they thought the program should be available for anyone with a cancer experience, and not offered only to those with sexual problems and fertility distress. The reasons for conducting an RCT before making the intervention available to unselected patient groups were explained (by the researchers) and accepted. Furthermore, we explained that if the intervention showed to be effective in reducing sexual problems and fertility-related distress, the goal was to collaborate with health care services to implement the program into regular care. Regarding the RCT testing, PRPs questioned why the Fex-Can intervention would be available only for study participants at baseline (1 year after diagnosis). They argued that problems could occur later during the planned follow-up period and suggested that the intervention should be made available to those with sexual problems or fertility distress in connection to late follow-ups (3 and 5 years after diagnosis). This promising suggestion will be taken into consideration in the RCT phase of the project. Other issues of value for implementation were identified, and it may be possible to address them throughout the development process (eg, ways to avoid dropout).

#### Input on Outcome Measures

The questionnaire measuring fertility distress, Reproductive Concerns After Cancer [31], which was one of the primary outcomes of the Fex-Can program, was discussed with the PRPs. Several items raised concern among the significant others about the risk of evoking worries, especially among participants in young ages (16-17 years). Based on this, we conducted additional cognitive interviews with 4 adolescents who had been treated for cancer to verify the acceptability of the measure among the youngest respondents.

## Discussion

### Principal Results

This study aimed to describe a co-creative process in the development of a self-help Web-based intervention to alleviate sexual problems and fertility-related distress in young cancer patients. The ultimate goal for patient and public involvement in research is to get another perspective on research projects by taking the PRPs’ lived experience into account [11] and getting new insights [32], which will result in more relevant interventions. We believe that our collaboration with 12 PRPs accomplished this goal and that both the demands of the PRPs and the needs of the study have been met in the development of the intervention, as stressed by van Gemert-Pijnen et al [10]. The input from the PRPs contributed to making the content of the Fex-Can intervention meaningful, relevant, and understandable. Furthermore, PRPs addressed the importance of an inclusive, diverse imagery and of creating a professional layout and persuasive design, and to improve support systems included in the intervention. The PRPs also affected the research project on an overall level by participating in naming the project, suggesting changes in the follow-up, and by questioning the implementation plan.

The co-creative process comprises bringing together researchers and stakeholders, exchanging ideas, and interacting to improve research [11]. This project is unusual as it from the start aimed to establish a long-term collaboration over 5 years, with a PRP group of considerable size in contrast to the more common set-up with multiple participants at a single event [15,16] or one or two participants on multiple occasions [9].The recruitment procedure [13,33] proved to be successful, with high attendance at meetings and only one person dropping out from the collaboration. The PRPs themselves described that their main motive for commitment in the project was a wish to help others in a situation similar to the ones they had experienced themselves when diagnosed with cancer. They also expressed that they appreciated sharing their experiences with people in the same age group, as described in other studies of young cancer patients [34].

We believe that our careful preparation and set-up of the collaboration created beneficial circumstances for PRPs to have a real impact on the project [12,32] for four main reasons. First, we allocated one person in the research group for all contacts with the PRPs and invested time getting to know each other. This procedure contributed to an environment that facilitated a positive and committed collaboration that may be especially important for younger PRPs, who otherwise might be too shy to express their opinion in a group [13]. Second, the involved researchers early on reflected on and discussed their expectations and perceptions regarding the roles of the PRPs in the project. We expected that a successful collaboration would require commitment, openness, and flexibility in the research group, where the PRPs were seen as experts on the patient perspective. However, this did not imply the incorporation of all PRP ideas. In cases when there was disagreement between researchers and PRPs, the researchers argued their points and clarified their view in order to achieve a common standpoint. Third, we compensated the PRPs for their time and expenses and also served food and drinks, which has been reported to increase PRPs’ feelings of being important for the project [33]. Fourth, in contrast to the common procedure when researchers are solely responsible for decisions on forms of collaboration [10], researchers and PRPs reached a common agreement on how meetings were to be organized in the Fex-Can study, even though the researchers decided on the agendas.

One of the main advantages of the long-term co-creative process was the possibility to, in an iterative way, fine-tune the Internet intervention through the PRPs’ repeated feedback on aspects that had been adapted following their earlier suggestions. The collaboration with PRPs was also timesaving for the researchers. Prompt confirmation of ideas and solutions that were well functioning enabled further development of these without delay, and weaker solutions could likewise be adjusted or discarded promptly. This made the researchers more confident in their work, and fewer parallel versions of layouts and contents had to be produced when existing versions were approved by the PRPs. Furthermore, when the PRPs saw that their feedback was incorporated in the program, they expressed satisfaction that their impact was valued, which further increased their motivation to participate. Another advantage of such a long-term collaboration was that the PRPs were able to contribute more as their understanding of the intervention’s intention grew. In addition, the researchers and the PRPs became more equal partners over time, and the researchers relied more on the PRPs who became indispensable in the project.

### Limitations

This study is novel in many ways and some methodological limitations should be mentioned. A problem with a long-term collaboration might be, as has been discussed elsewhere [35,36], that the PRPs become “professional” patients, who incorporate the researchers’ views and alienate themselves from the target group. As the relationship between researchers and patients is asymmetrical [35], patients’ experiential knowledge and input might also be unintentionally overruled [37]. However, the PRPs were encouraged to express their opinions during the meetings and all PRPs did so, including expressing divergent opinions and questioning ideas from the researchers. The PRPs often referred to how they would have reacted 1 year after their cancer, clearly identifying themselves with future users of the intervention. The meetings were held 2-3 times per year and the PRPs outnumbered the researchers in all discussions, limiting the risk that PRPs became “professional” patients. An opposite problem might be that researchers let the PRPs’ ideas and suggestions run the project without being critical. However, we strived to create a balance between new ideas and methods known to be effective.

Further, the PRPs were not recruited based on their level of problems in the areas targeted in the intervention (ie, sexual problems and fertility distress). Therefore, it was not known if individuals with high levels of problems/distress were adequately represented, thus possibly limiting representativeness. We are also aware of the lack of heterogeneity in the PRP group, as most of them were well educated and there seemingly was a lack of diversity in ethnicity and sexual orientation. The characteristics of the PRPs may therefore have contributed to less variety in expressed opinions. However, the perspectives of different groups of future users were often brought up by the PRPs, who argued that the intervention must suit users with, for example, different sexual orientations. Furthermore, the research team’s composition was mixed regarding country of birth and sexual orientation, which may to some degree have broadened the perspectives represented. In other aspects the PRP group was heterogeneous in that it included men and women of different ages, both singles and those living with a partner, from different parts of the country. An important limitation is, however, that this study is based on the researchers’ views of the co-creative process, with only indirect reports from the PRPs and no independent assessment of the collaboration process.

When individual PRPs expressed conflicting viewpoints the researchers sometimes found it difficult to know whom they should listen to. There is a risk that those who talk loudly or eloquently receive more attention or that researchers pay attention to comments that are close to their own opinions. We tried to avoid this in several ways, for example, by letting PRPs give feedback on the mock-up and early versions of the website confidentially by writing comments online and by discussing topics in small groups to facilitate expression of opinions. Another way of equalizing power relations between the collaborating parties was to strive for researchers to be in the minority in all discussions. Finally, several of the researchers had training in counseling and were used to encouraging other persons, which we believe contributed to a sensitive and constructive discussion climate at collaboration meetings.

### Conclusion

A long-term collaboration between researchers and a committed group of patient research partners contributed substantially to the development of a self-help Web-based intervention. With suggestions and continuous feedback from PRPs, it was possible to develop a Web-based intervention believed to be relevant and attractive for young persons with cancer having sexual problems or fertility distress. The collaboration with PRPs will continue in the following steps of testing the Fex-Can intervention. The intervention will first be tested in a feasibility study with cancer patients, where PRPs will participate in the interpretation of results. The effectiveness of the intervention will thereafter be tested in an RCT targeting a nationwide cohort of adolescents and young adults with problems and distress related to sexuality and fertility after cancer.

## Abbreviations

Fex-Can: fertility and sexuality following cancer
PRP: patient research partner
RCT: randomized controlled trial
